# Use the rating of perceived exertion to evaluate load in collegiate male soccer player, validity and influencing factors

**DOI:** 10.1038/s41598-025-01942-y

**Published:** 2025-05-15

**Authors:** Zheng Li, ShengLei Qin, XiaoTian Li, DingMeng Ren

**Affiliations:** 1 China Football College, Beijing Sport University, Beijing, China; 2 APAC Team, Catapult Sports, Melbourne, Australia; 3 Physical Education Department, Central South University, Changsha, China; 4 School of Sport Training, Wuhan Sports University, Wuhan, China

**Keywords:** SRPE, Training load, Soccer, Well-being, Heart rate, Health care, Physics

## Abstract

The aim of this study was to evaluate the efficacy of session rating of perceived exertion (SRPE) in collegiate male soccer players and to identify the factors that influence SRPE, with the aim of developing a predictive model. Fifteen Chinese collegiate soccer players (age 19.6 ± 0.8 y, height 179.1 ± 5.4 cm, weight 70.8 ± 4.9 kg, body mass index 22.1 ± 1.7 kg/m^2^, body fat 10.9 ± 2.3%, heart rate rest 56.6 ± 8.0, heart rate max 194.9 ± 7.3) participated in the study. Global position system (GPS) wearable devices, pre-session well-being questionnaire [sleep, fatigue, delayed onset muscle soreness (DOMS), stress and energy] and SRPE was carried out to collect training load (TL) and well-being variable, 977 observations from training and competition during 7 months were recorded. Moderate to very large correlations were found between SRPE and heart rate-based load Banister TRIMP (0.58 ± 0.14, 0.38 ≤ r ≤ 0.78, *p* < 0.05) and Edwards TL (0.59 ± 0.14, 0.37 ≤ r ≤ 0.83, *p* < 0.05). The stepwise multiple regression show that in all courses, total distance (TD), fatigue, Edwards TL, sleep, sprint distance (> 24 km/h), inertial movement analysis (IMA) can explain 32.4% of the adjusted R^2^ of SRPE (y = − 152.672 + 0.034 TD + 49.101 Fatigue + 0.834 Edwards TL + 36.012 sleep + 0.538 SD + 0.871 IMA), and the variance inflation factors are 2.470, 1.011, 2.386, 1.011, 1.181, 1.143 respectively. Moderate to very large correlations demonstrate that SRPE is an effective method for quantifying collegiate male soccer players. Multiple regression model showed that internal load, external load and pre-training well-being status affect SRPE, demonstrating that SRPE is a global method for assessing internal load in collegiate male soccer players.

## Introduction

Accurately quantifying the load during sports training is of great significance to understand whether players have the corresponding potential, adapt to the training plan, and respond to the training plan^1,2^.It is also important for understanding players’ recovery needs, assessing their fatigue status, and minimizing the risk of nonfunctional overreaching, injury, and disease^1,2^. Understanding and managing training load is essential for optimizing performance and safeguarding player health.

Measuring training load can be categorized as internal and external. Internal training load is the relative biological (both physiological and psychological) stressors imposed on the player during training or competition^1^. External training load is an objective measure of the work a player does during training or competition and is assessed independently of internal workload^1^. Internal and external loads are interdependent and provide valuable insights into the recovery status of soccer players^3^. Among the methods for quantifying internal load, the session rating of perceived exertion (SRPE) stands out for its simplicity and practicality^4^. Since its introduction, numerous studies validated the effectiveness of SRPE in measuring internal load across different populations and sports^5^. Although internal load determines the results of training^6^, its evaluation is challenging due to the complex interplay of multiple factors^7^. Nonetheless, the SRPE method is recommended as the primary global measurement of internal load^8^.

Simple methods (e.g., SRPE) remain widely applicable. However, advances in microtechnology (e.g., heart rate monitors, GPS, accelerometers) now enable detailed measurements of both external load (EL) and internal load (IL) during team sports training^9^. While EL measures objective workload, IL reflects players’ physiological and psychological responses, which can vary significantly even under identical EL conditions due to factors like fatigue, mood, training history, or illness^1^. Recent research highlights the relationship between EL and IL, showing that this relationship depends on measurement methods and training modes. To better understand players’ responses to cumulative training stress and evaluate recovery needs, practitioners increasingly recommend combining EL and IL monitoring for a more comprehensive assessment of training status and performance^3,6,7^.

Although various external load metrics lead to data overload, they provide new insights into the relationship between internal and external loads, allowing us to consider external load factors that influence SRPE. Multiple studies demonstrated that SRPE in soccer correlated well with internal load indicators based on heart rate (e.g., TRIMP) and shows a clear relationship with various EL metrics (e.g., total distance, PlayerLoad, distance in specific speed threshold, etc.)^10–13^. Previous models for SRPE, a partial correlation model showed that high-speed distance (m), impacts (n), and accelerations (n) were predictors of RPE-TL(partial correlation with 0.114, 0.451 and 0.371 respectively)^14^, and a generalized estimating equation showed that PlayerLoad, high-speed running distance , and distance in acceleration were predictors of RPE (estimate with 0.6328, 0.0965 and 1.1157 respectively)^12^. However, the former study involved players from the Premier League and the latter from youth national teams. Subjects’ expertise-level characteristics can influence RPE^5^, and the varying expertise-levels and experience with the use of RPE scales limit the generalization of results obtained from senior players^13^.

Among the factors influencing SRPE, in addition to internal and external loads, athletes’ well-being status continues to draw the attention of researchers^13^. For instance, a mixed-effects model for senior players revealed that a reduction in well-being (muscular soreness, sleep quality, fatigue, stress and energy level) Z-score negatively affected their ability to complete high-speed and maximal-speed distances^15^. However, a separate study using machine learning suggested that well-being questionnaires had poor predictive power for both IL and EL, emphasizing the need for caution when interpreting and applying these responses^16^. At the same time, collegiate players may have additional stressors such as multiple athletic obligations, schooling, social interactions, and physical maturation^17^. The reality is that as training and competition increase, these players participate in considerable rigorous training volumes due to their participation in several sports^1^. Many players are not just committed to one sport and/or team, they may compete on school teams and clubs or varsity teams in one or more sports, while also exercising regularly through school physical education classes^17^. Additional stress on collegiate players may cause them to have different pre-training well-being status and affect their reported RPE.

Under this circumstances, the first aim of this study is to investigate the validity of SRPE in collegiate soccer players,the hypothesis is that SRPE is an effective method for assessing the internal load of collegiate male soccer players, with a clear correlation between SRPE and heart rate-based internal load. Secondly, the aim is using internal load, external load and well-being status to build a model to predict SRPE. The hypothesis is that internal/external load and well-being variables have a significant impact on SRPE.

## Methods

### Participants

Considering statistical power, we set the effect size *r* to 0.5 (large correlation), *f*^2^ to 0.15 (moderate effect), the significance level to 0.05, and the statistical power to 0.8. Using RStudio (Version 2024.09.1 + 394, https://posit.co/products/open-source/rstudio/), it was calculated that a minimum of 29 samples are required for correlation analysis, while stepwise multiple regression requires 114 samples.

Fifteen Chinese collegiate male soccer players (age 19.6 ± 0.8 y, height 179.1 ± 5.4 cm, weight 70.8 ± 4.9 kg, BMI 22.1 ± 1.7 kg/m^2^, body fat rate 10.9 ± 2.3%, HR_rest_ 56.6 ± 8.0, HR_max_ 194.9 ± 7.3) participated in the study. All athletes met the following inclusion criteria: (i) over ten years of systematic training experience; (ii) no major injuries in the past six months; (iii) long-term participation in monitoring. They conducted a total of 4–6 training/competition activities per week, with each training/competition duration ranging from 60 to 180 min. We obtained the informed consent of all participants. All participants were informed of the potential risks and benefits before conducting this test and voluntarily participated in this study. All experimental procedures met the requirements of ethical review and were conducted in accordance with the Declaration of Helsinki. The Ethics Review Board of Wuhan Sports University also approved the conduct of this study (approval number: 2023070).

### Experimental design

**Fig. 1:**
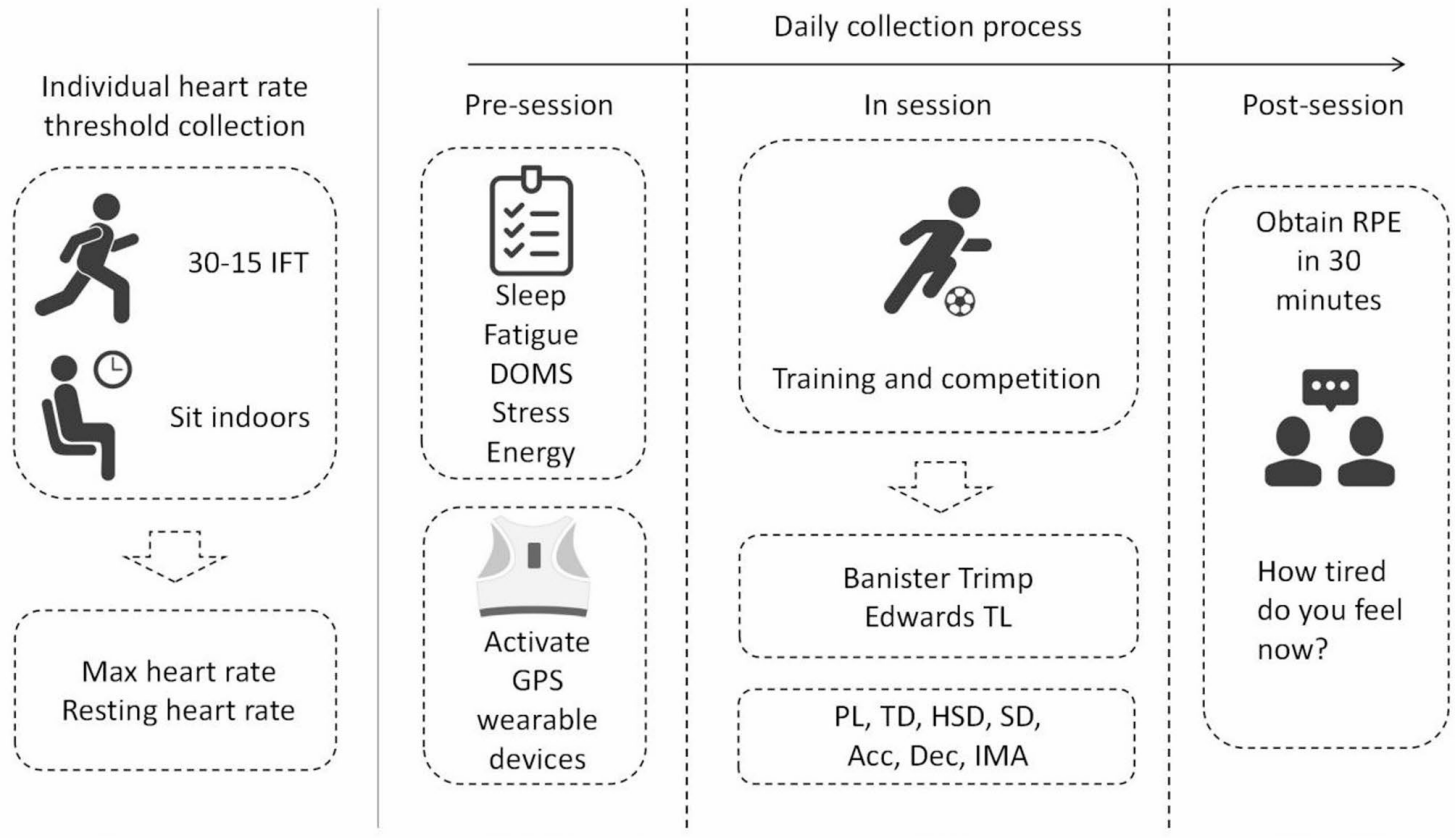
Experimental procedure.

Our experimental design, illustrated in Fig. 1, includes four components: measuring individual heart rate thresholds, collecting pre-session well-being status, monitoring in session, and recording RPE values after training. The data includes training and competitions.

30-15IFT: 30–15 intermet Intermittent fitness test; PL:PlayerLoad (AU); TD: total distance (m); HSD: high speed distance (m, > 21km/h); SD: sprint distance (m, > 24 km/h); Acc: acceleration effort (n, > 3.5 m/s^2^); Dec: deceleration effort (n, > 3.5 m/s^2^); IMA: inertial movement analysis effort (n, Acc + Dec + CoD Right + CoD Left, > 3.5 m/s^2^).

Before the monitoring started, we first used InBody 770 (InBody 770, South Korea) to measure the players’ basic information (height, weight, body fat rate). In order to obtain resting heart rate, we used Polar Team Pro (Polar Team Pro, Finland) to collect players’ resting heart rate while sitting indoors. The participants were required to be awake and quiet throughout the entire process, and the average heart rate of 5 min was taken as the resting heart rate. To obtain maximum heart rate, we performed the 30–15 IFT test, which has been proven to be a reliable method of inducing maximum heart rate in players^18^. Players are asked to complete this test to the best of their ability.

Based on previous recommendations^19,20^, we conducted a well-being questionnaire before each training session to assess player well-being status. The well-being questionnaire contains five questions, sleep quality, fatigue, DOMS, stress and energy. Scores of 1–7, sleep quality and energy with 1 and 7 representing poor and very good wellness ratings, fatigue, DOMS, stress are the opposite, 1-point increments. Questionnaires were distributed 30 min before each session.

We used the GPS wearable device Catapult Vector S7 to measure internal and external loads during training/competition. It can monitor satellite positioning GPS (10 Hz GPS) and local positioning information, inertial sensor and heart rate data (from signal measured by ECG). When using it, wear a vest and fix it to the middle of the shoulder blades on the back. Use the OpenField (version 3.10.0, https://cn.openfield.catapultsports-cn.com) provided by Catapult company to edit data. We assigned each participant an independent GPS module to reduce unnecessary errors.

Based on the internal load of heart rate, we use two TRIMP algorithms, Banister TRIMP^21^ and the Edwards TL^22^. It has been proven applicable across various training modes^23^. The Banister TRIMP calculation method follows:

$${{BanisterTRIMP}}\, = \,{{Duration}}\left( {{{min}}} \right)\, \times \,\left( {\Delta {{HR ratio}}} \right)0.{{64e}}^{{{{b}}(\Delta {{HR ratio}})}}$$

∆HR ratio = (HRex − HRrest)/(HRmax − HRrest), b = 1.92 for males, where the HRex is the average HR during session, HRrest is the the average heart rate during rest. The calculation method by Edwards TL involves assigning constants (1, 2, 3, 4, 5) multiplied by the time (in minutes) spent in specific heart rate zones (e.g., 50–60%, 60–70%, 70–80%, 80–90%, 90–100% of maximum heart rate), summed up the results and expressed in arbitrary units (AU).

According to previous related studies^12,14^, our external load predictors include: PlayerLoad (AU), total distance (m), high speed distance (HSD, m, > 21km/h), sprint distance (SD, m, > 24km/h), acceleration effort (Acc, n, > 3.5m/s^2^), deceleration effort (Dec, n, > 3.5m/s^2^), inertial movement analysis effort (IMA, n, Acc + Dec + CoD Right + CoD Left, > 3.5m/s^2^).

Based on the CR-10 scale developed by Foster et al.^24^, in view of the nationality characteristics of the participants and the limitations of translation efficiency, we used the scale adapted by Liu Hongyou et al.^25^ to obtain the RPE value. It is described as “how tired do you feel now”. RPE values were collected individually from players within 30 min after each session, and we did not inform their teammates or coaches of the RPE values to minimize errors. In order to explore the validity of SRPE in quantifying internal load, we calculated RPE × training/competition duration (in minutes) to obtain internal load in arbitrary units (AU).

Record the start time and end time of the training/competition in minutes, excluding speeches before the session and summaries after the session. Both the warm-up and half-time periods of competitions were included in this study. If players do not participate in the entire session due to training/competition injuries or other unexpected reasons, the data will not be included in this study. Data fragmentation and loss due to uncontrollable factors were excluded from the analysis. Only data that contained all three sections (fill out the well-being questionnaire before training, complete the training, and report RPE after training) were included in the analysis. After each session, the data of relevant variables will be imported into EXCEL for processing.

### Statistic

Data are expressed as Mean ± SD. Associations between variables were assessed using Pearson correlation coefficient. Magnitude of correlation coefficients were considered as trivial (r < 0.1), small (0.1 < r < 0.3), moderate (0.3 < r < 0.5), large (0.5 < r < 0.7), very large (0.7 < r < 0.9) and almost perfect (0.9 < r < 1.0)^26^. A stepwise multiple linear regression model was constructed to determine whether players’ SRPE could be predicted by well-being status and internal/external load. The goodness of our model was measured by the correlation coefficient(R^2^).We use collinearity diagnostic (VIF) to determine multicollinearity between independent variables. All data processing was performed using IBM SPSS Statistics (version 25, https://www.ibm.com/products/spss-statistics), with a significance value set at *p* < 0.05.

## Result

We completed training monitoring from September 2023 to May 2024, excluding rest season (January–February) and unreliable data due to uncontrollable factors. We collected a total of 977 records from 15 players, ranging from 41 to 83, meets the requirements for statistical power. The descriptive statistics of the internal load variables are shown in Table 1.

**Table 1:** Descriptive statistics of the variables.

IL	Mean ± SD	ET	Mean ± SD	Well-being	Mean ± SD
RPE(AU)	5.6 ± 1.9	PL(AU)	762.9 ± 227.8	Sleep	4.3 ± 1.1
SRPE(AU)	697.8 ± 298.3	TD(m)	6917.1 ± 2147.0	Fatigue	4.2 ± 1.2
BT(AU)	113.7 ± 51.1	HSD(m)	168.8 ± 175.3	DOMS	3.9 ± 1.4
ETL(AU)	231.0 ± 84.9	SD(m)	50.8 ± 75.0	Stress	3.5 ± 1.5
		Acc(n)	14.4 ± 9.7	Energy	4.5 ± 1.6
		Dec(n)	15.4 ± 9.7		
		IMA(n)	34.4 ± 23.0		

IL, internal load, EL, external load, BT, Banister TRIMP, ETL, Edwards TL, PL, PlayerLoad, TD, total distance, HSD, high speed distance (> 21 km/h), SD, sprint distance (> 24 km/h), Acc, acceleration (> 3.5 m/s^2^), Dec, deceleration (> 3.5m/s^2^), IMA, inertial movement analysis (acceleration + deceleration + CoD right + CoD left, > 3.5 m/s^2^).

Table 2 presents the correlations between SRPE and internal load (Banister TRIMP, Edwards TL) in 15 players across all observations, training sessions, and competitions.

**Table 2:** Relationships between SRPE and internal load variables.

	Total session			Training			Competition		
	BT	ETL	N	BT	ETL	N	BT	ETL	N
P1	–	0.44**	41	0.39*	–	30	–	0.88**	11
P2	0.44**	0.37*	44	0.47**	0.44*	30	–	–	14
P3	0.54**	0.59**	51	0.82**	0.89**	38	0.80**	0.67*	13
P4	0.74**	0.63**	51	0.61**	0.47**	43	0.96**	0.88**	8
P5	0.41**	0.46**	56	0.75**	0.49**	38	–	–	18
P6	0.38**	0.47**	70	0.52**	0.49**	51	–	–	19
P7	0.57**	0.70**	67	0.57**	0.61**	49	0.55*	0.80**	18
P8	0.69**	0.52**	63	0.65**	0.55**	43	0.55*	–	20
P9	0.40**	0.45**	73	0.49**	0.47**	52	0.46*	0.60**	21
P10	0.78**	0.82**	70	0.83**	0.87**	49	0.63**	0.70**	21
P11	0.67**	0.69**	75	0.61**	0.59**	56	0.49*	0.54*	19
P12	0.60**	0.65**	72	0.61**	0.62**	55	0.62**	0.70**	17
P13	0.78**	0.83**	81	0.59**	0.69**	56	0.65**	0.76**	25
P14	0.56**	0.59**	80	0.50**	0.51**	58	0.44*	0.49*	22
P15	0.50**	0.66**	83	0.60**	0.65**	60	0.47*	0.70**	23
Max	0.78**	0.83**	83	0.83**	0.89**	60	0.96**	0.88**	25
Min	0.38**	0.37**	41	0.39**	0.44**	30	0.44**	0.49**	8
Mean ± SD [95%]	0.58 ± 0.14 [0.50–0.57]	0.59 ± 0.14 [0.52–0.67]		0.60 ± 0.12 [0.53–0.67]	0.59 ± 0.14 [0.51–0.68]		0.60 ± 0.16 [0.50–0.70]	0.70 ± 0.12 [0.62–0.79]	

BT, Banister TRIMP, ETL, Edwards TL.

**p < 0.01; *p < 0.05; –: no significant relationship (*p* > 0.05).

Table 3 covers the effects of well-being status, internal and external loads on SRPE, separately and together. In all sessions, TD, fatigue, Edwards TL, sleep, SD, IMA had significant impact on SRPE (R^2^ changes were 0.234, 0.030, 0.026, 0.018, 0.016, 0.004), Edwards TL, sleep, fatigue, PL, SD significantly affected training (R^2^ changes were 0.175, 0.047, 0.047, 0.022, 0.013), while TD, fatigue, IMA significantly influenced matches (R^2^ changes were 0.257, 0.032, 0.028).

**Table 3:** The influence of well-being and internal/external load over SRPE taking into account training sessions and competitions both separately and together.

Dependent Variable	SRPE					
Variables	ALL Sessions	P	95%CI		Beta	VIF
Adjusted R^2^	0.32					
Constant	− 152.67	0.004	− 255.06	− 50.29		
TD	0.03	< 0.001	0.02	0.05	0.25	2.47
Fatigue	49.10	< 0.001	35.98	62.22	0.19	1.01
Edwards TL	0.83	< 0.001	0.55	1.11	0.24	2.39
Sleep	36.01	< 0.001	22.24	49.79	0.14	1.01
SD	0.54	< 0.001	0.31	0.76	0.14	1.18
IMA	0.87	0.017	0.16	1.59	0.07	1.14

Variables	Training	P	95%CI		Beta	VIF
Adjusted R^2^	0.30					
Constant	− 204.66	< 0.001	− 318.70	− 90.61		
Edwards TL	1.08	< 0.001	0.78	1.38	0.31	1.89
Sleep	53.80	< 0.001	39.45	68.15	0.23	1.02
Fatigue	46.20	< 0.001	32.63	59.78	0.21	1.01
PL	0.24	< 0.001	0.13	0.35	0.18	1.90
SD	0.50	< 0.001	0.23	0.77	0.12	1.09

Variables	Competition	P	95%CI		Beta	VIF
Adjusted R^2^	0.31					
Constant	63.52	0.44	− 97.16	224.20		
TD	0.06	< 0.001	0.04	0.07	0.42	1.17
Fatigue	54.31	0.001	23.55	85.07	0.18	1.01
IMA	2.24	0.001	0.91	3.56	0.18	1.16

PL, PlayerLoad, TD, total distance, SD, sprint distance (> 24 km/h), IMA, inertial movement analysis (acceleration + deceleration + CoD right + CoD left, > 3.5 m/s^2^).

## Discussion

Considering the limited research on the application of SRPE in collegiate soccer players, the purpose of this study was to test the validity of SRPE in assessing the internal load of collegiate soccer players, and to predict the players’ SRPE based on the players’ well-being status before training/competition, completed external load output, and endured internal load. The study results indicated that SRPE was highly correlated with Banister TRIMP and Edwards TL, making it an effective tool for assessing internal load in collegiate soccer players. The regression model revealed that well-being status (fatigue, sleep), external load (total distance, SD, IMA, PlayerLoad), and internal load (Edwards TL) influenced players’ SRPE (adjusted R^2^: 29–32%), further demonstrating that SRPE serves as a global indicator of load.

The first finding of the study revealed a moderate to very strong correlation between SRPE and heart rate-derived internal training load. Previous studies on youth athletes reported that the individual correlations between SRPE and heart rate-based internal load ranged from 0.50 to 0.85^27^. In professional soccer players, the correlations with Banister TRIMP and Edwards TL were 0.73 and 0.77^28^. In female players, SRPE was also highly correlated with Edward TL and Bannister TRIMP (0.70 ≤ r ≤ 0.90)^29^. Similarly, SRPE demonstrated strong correlations with heart rate-based load across different training modalities^30^. Despite slight differences in statistical methods and training models, our findings were consistent with previous research^10,27–31^, suggesting that the SRPE method is suitable for collegiate players. In addition, our research results also showed that the correlation between SRPE and heart rate load for some players during competition was not significant. In fact, due to the complex situational and environmental factors^32^, heart rate-based assessments have limitations in competitions, while SRPE accounts for physiological and psychological factors^8^, possibly weakening their correlation.

Although internal load determines the functional outcomes of a training session^6^, the influence of multiple factors makes it difficult to quantify using a single method^7^, requiring consideration of the combined physiological and psychological responses^4^. The regression model we constructed showed that internal load (Edwards TL), external load (distance, SD, IMA, PlayerLoad) and health status (fatigue, sleep) can explain 32% of the adjusted variance of SRPE. Interestingly, we found that Edwards TL influenced SRPE, while Banister did not. Both heart rate-based load measures assessed athletes’ internal load and showed an almost perfect correlation (r = 0.98) in previous studies^28^. Recent studies found that RPE could classify the percentage of maximum heart rate during women’s soccer training^31^, with similar results observed in men’s ice hockey^33^. It appears that heart rate percentage-based load has a stronger association with RPE, but further research is needed.

Our study indicated that, among external load measures, total distance has the greatest impact on SRPE (R^2^ changes was 0.234). Our findings are similar to previous research that showed total distance significantly affects SRPE in rugby league players^9^ and elite youth soccer players^12^. At the same time, the results we obtained are identical to those of a meta-analysis, which showed that total distance is the indicator most closely related to internal load and intensity^7^. We conclude that total distance metrics elicit similar responses in the training of student-players as do professional and elite players. In the absence of relevant GPS equipment, SRPE can provide assistance for training monitoring of collegiate players. Our study also showed that PlayerLoad significantly influences SRPE during training, consistent with previous findings in team sports^9,12^. PlayerLoad accounts for the total stress generated by accelerations, decelerations, and changes in direction^9^. Our findings highlighted the importance of mechanical load in internal responses^34,35^.

Meanwhile, our study showed that running distance at high-speed thresholds also influences athletes’ SRPE. Although the speed threshold differed from previous research on elite soccer players (> 19.8 km/h)^12^ and rugby league players (> 14.4 km/h)^14^, we also found that distance run at sprint speed (> 24 km/h) affected SRPE in collegiate players. When investigating the physical demands of soccer training and competition, there is often an emphasis on distance covered at high speeds^36^. It is important to note that this study utilized general thresholds rather than individualized ones. Considering the skill level of the participants, they may experience higher internal loads at the same speeds^37^. Furthermore, individualized speed thresholds appear to be more applicable for injury prevention in athletes^38^. We recommend using personalized speed thresholds to better align with the participants’ age, skill level, and other characteristics^37^.

At the same time, changes in acceleration indicators are also important for studying the physical needs of soccer players. Soccer involves many non-periodic changes characterized by changes in acceleration that increase the physical demands on the player even when running within low speed thresholds^14^. Acceleration activity is associated with mechanical stress and increases metabolic demands^35^ and energy expenditure^39^. Previous research with repeated sprint exercises at different accelerations showed that maximal acceleration produced higher internal loads related to heart rate than submaximal acceleration^40^. Our research showed that, in all sessions, IMA was one of the influencing factors for collegiate players’ SRPE. IMA efforts involve sharp accelerations and decelerations as well as changes of direction, which means high eccentric muscle effort and high energy costs^41^. In our study, IMA included high-intensity (> 3.5 m/s^2^) acceleration, deceleration, changes to the left, and changes to the right. This is similar to previous research on elite^12^ and professional^14^ level soccer players, where acceleration was a significant influence on SRPE. This emphasized the importance of high-intensity acceleration changes in soccer and that acceleration affects SRPE in collegiate players as it does in elite-level soccer players.

In addition, we also found that across all sessions, pre-training sleep and fatigue status of collegiate soccer players affected SRPE. A previous section of the study analyzed the relationship between players’ pre-training well-being and subsequent internal and external load. Previous research showed that the less muscle soreness reported by American collegiate football players before training (a one-unit increase in Z-score), the lower the internal load SRPE was by approximately 4.4% (90% CI − 8.4, − 0.3; SMD: − 0.05)^42^. A study involving collegiate male soccer players showed that morning fatigue negatively predicted SRPE (γ10 = − 66.8, *p* = 0.003) after afternoon training^43^. These findings appeared to contradict our results, but it was important to note that the model analysis method and the fatigue questionnaire (Overall Fatigue Scale, 0 = no fatigue; 5 = strong fatigue; 10 = maximal fatigue) we used were different^43^. At the same time, the findings of this study were accompanied by a reduction in external load (TD, PL, IMA, RHIE) and the GPS variables were not controlled, which made it difficult to compare the study results.

Studies showed that sleep duration the previous night could positively predict the external load output of soccer players the next day^43^. A linear mixed-effects model of American collegiate football players showed that sleep was not related to SRPE, whether modeled with all health variables or individually^42^. Our study showed that across all sessions, sleep was one of the predictors of SRPE, and for every unit increase in sleep (better sleep quality), SRPE increased by 36.01 units. But what we need to note is that the scales we used are different (5 scales and 7 scales). In another study, sleep duration the previous night predicted greater TD, HSD, and RHIE output during training^43^. Better sleep quality/quantity may induce greater external load output, thereby affecting the SRPE achieved. However, comparisons are difficult to make without quantifying the specifics of external loads. Nonetheless, our regression equation showed that sleep had a clear impact on SRPE.

Our study provides a reference for understanding the relationship between internal and external loads in collegiate soccer players. It demonstrates that SRPE is an effective tool for assessing internal load in this population, which can be particularly useful for teams lacking monitoring equipment. Coaches should also consider the various factors influencing SRPE when designing training programs.

However, our study has certain limitations. A more precise classification of training types could offer more detailed insights, and we recommend using individualized speed zones to better match the characteristics of participants, such as age and training level. Future research should aim to incorporate technical and tactical contextualization and analyze their impact on task design.

## Conclusion

The findings provide practitioners with guidance on effectively using SRPE, confirming it as a valid measure of internal load in collegiate soccer players. Our regression model indicates that external/internal load and pre-training well-being influence SRPE, Coaches should consider the influence of multiple factors when using SRPE.

## Data Availability

The data collected in this study are not publicly available but can be obtained from the authors on reasonable request. Contact the author Zheng Li, Email: 2908036617@qq.com.
